# Remote Sensing Extraction of Damaged Buildings in the Shigatse Earthquake, 2025: A Hybrid YOLO-E and SAM2 Approach

**DOI:** 10.3390/s25144375

**Published:** 2025-07-12

**Authors:** Zhimin Wu, Chenyao Qu, Wei Wang, Zelang Miao, Huihui Feng

**Affiliations:** School of Geosciences and Info-Physics, Central South University, Changsha 410083, China; wuzhimin@csu.edu.cn (Z.W.); chenyaoqu@csu.edu.cn (C.Q.); zelang.miao@csu.edu.cn (Z.M.); hhfeng@csu.edu.cn (H.F.)

**Keywords:** damaged building extraction, YOLO-E, Segment Anything Model 2 (SAM2), multi-source remote sensing, earthquake damage assessment

## Abstract

In January 2025, a magnitude 6.8 earthquake struck Dingri County, Shigatse, Tibet, causing severe damage. Rapid and precise extraction of damaged buildings is essential for emergency relief and rebuilding efforts. This study proposes an approach integrating YOLO-E (Real-Time Seeing Anything) and the Segment Anything Model 2 (SAM2) to extract damaged buildings with multi-source remote sensing images, including post-earthquake Gaofen-7 imagery (0.80 m), Beijing-3 imagery (0.30 m), and pre-earthquake Google satellite imagery (0.15 m), over the affected region. In this hybrid approach, YOLO-E functions as the preliminary segmentation module for initial segmentation. It leverages its real-time detection and segmentation capability to locate potential damaged building regions and generate coarse segmentation masks rapidly. Subsequently, SAM2 follows as a refinement step, incorporating shapefile information from pre-disaster sources to apply precise, pixel-level segmentation. The dataset used for training contained labeled examples of damaged buildings, and the model optimization was carried out using stochastic gradient descent (SGD), with cross-entropy and mean squared error as the selected loss functions. Upon evaluation, the model reached a precision of 0.840, a recall of 0.855, an F1-score of 0.847, and an IoU of 0.735. It successfully extracted 492 suspected damaged building patches within a radius of 20 km from the earthquake epicenter, clearly showing the distribution characteristics of damaged buildings concentrated in the earthquake fault zone. In summary, this hybrid YOLO-E and SAM2 approach, leveraging multi-source remote sensing imagery, delivers precise and rapid extraction of damaged buildings with a precision of 0.840, recall of 0.855, and IoU of 0.735, effectively supporting targeted earthquake rescue and post-disaster reconstruction efforts in the Dingri County fault zone.

## 1. Introduction

On 7 January 2025, a devastating magnitude 6.8 earthquake jolted Dingri County in Shigatse City, Tibet. The event caused severe damage to infrastructure, with many public and residential buildings either partially damaged or entirely destroyed. Accurate and rapid extraction of damaged buildings in the aftermath of an earthquake is of paramount importance [1,2]. During emergency rescue efforts, rapid extraction of damaged areas allows responders to locate potential survivor sites more efficiently. This prioritization supports better allocation of personnel and supplies to the most critical zones [3,4]. In the reconstruction phase, damage assessment data serve as a foundation for urban planning, helping authorities select safer locations for rebuilding and make informed decisions throughout the recovery process [5,6,7].

Traditional methods to extract damaged buildings depend on manual field surveys [8,9]. These methods take much time and effort and also pose surveyors at risk, especially in areas with rough terrain or aftershocks [10,11]. Advances in remote sensing and computer vision now allow automated methods [12,13]. These methods utilize satellite and aerial imagery not only as effective alternatives to manual interpretation, but also as powerful tools for large-scale monitoring [14,15,16,17,18]. Recent advancements in change detection techniques have significantly improved the automatic extraction of earthquake-induced building damage using remote sensing imagery. By comparing pre- and post-event images, traditional pixel-based differencing methods have been largely replaced by object-based and deep learning approaches, such as Siamese networks and encoder decoder architectures (U-Net and Transformer variants), which offer higher accuracy and robustness against noise and illumination variations, as reviewed by Gu et al. (2024) [19]. Ramadhan et al. (2024) [20] proposed a feature-concatenated Siamese neural network for building damage assessment. Recent studies, such as Yu et al. (2025) [21], introduced a dual-branch Transformer model that leverages multi-scale features and self-attention to detect subtle damage patterns.

Although the above studies highlight the capability of change detection methods in post-disaster building damage extraction, for emergency response purposes, it remains challenging to obtain pre- and post-disaster corresponding imagery in a timely and synchronized manner. This is particularly problematic in remote, inaccessible, or cloud-covered regions [22]. In addition, discrepancies in acquisition time, spatial resolution, or viewing angle between the images can further introduce false positives and missed detections. As a result, recent research has increasingly focused on single-date damage detection methods that do not rely on both pre-disaster and post-disaster imagery. For instance, Hong et al. (2022) [23] introduced EBDC-Net, a deep convolutional neural network for building damage classification. It uses only post-disaster aerial images. The model combines semantic feature extraction and a damage-aware classification module. It achieves 94.4% accuracy in binary classification and 77.5% in multi-class damage levels. This shows strong potential for single-date methods. Similarly, Hacıefendioğlu et al. (2024) [24] evaluated several semantic segmentation models—including U-Net, FPN, and PSPNet—on satellite images from the 2023 Türkiye earthquakes, all of which were trained and tested solely on post-event data. Their results showed consistently high performance, with all models exceeding 96% accuracy and U-Net achieving the best balance between recall and IoU. To further enhance the generalizability of such methods across different disaster scenarios, Zheng et al. (2024) [25] proposed a self-training change-aware adaptation framework (STCA), which allows pre-trained models to adapt to new disaster events using only unlabeled post-disaster imagery. This approach eliminates the need for pre-event or labeled data in the target domain, making it highly suitable for rapid, large-scale emergency deployments.

Among various models, YOLO-E (Real-Time Seeing Anything), as the latest lightweight segmentation-enhanced version of the YOLO series, demonstrates high-speed and accurate object segmentation performance [26]. Its ability to swiftly locate targets within large-scale satellite imagery is particularly advantageous for the timely extraction of potentially damaged buildings in earthquake-affected areas. SAM2 (Segment Anything Model 2) is a cutting-edge tool for fine-grained, pixel-level segmentation [27]. It excels at delineating object boundaries with high precision, making it well-suited for distinguishing damaged structures from surrounding features and undamaged buildings. However, when applied independently, each model exhibits limitations in handling complex post-disaster scenes [28,29].

Therefore, this study aims to fill this gap by integrating YOLO-E and SAM2 into a hybrid model in the order of processing. By leveraging the unique strengths of these two models, we expect to develop a more efficient and accurate method for extracting damaged buildings in the Shigatse earthquake area, providing valuable support for both emergency response and long-term reconstruction efforts.

## 2. Data and Methods

### 2.1. Study Area

**Figure 1:**
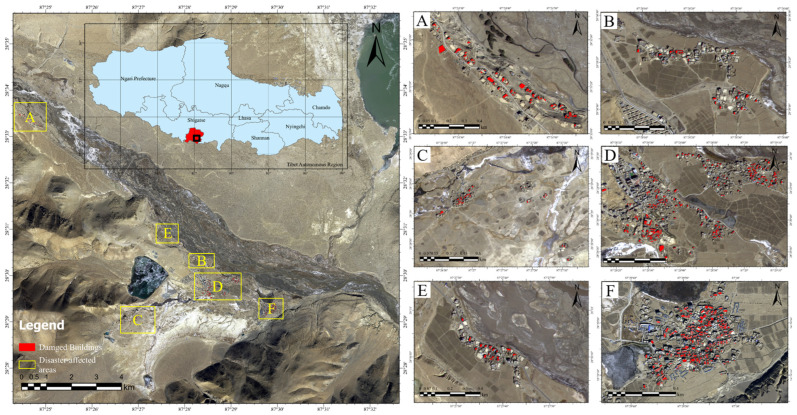
Overview of the study area in Shigatse, Tibet Autonomous Region, China. The left main panel shows the general locations of the disaster-affected regions (**A**–**F**), outlined in yellow. The right subpanels (**A**–**F**) present close-up views of the six disaster-affected areas, with red polygons indicating damaged buildings.

As shown in Figure 1, the study area is located in Dingri County, Shigatse City, Tibet Autonomous Region. It is located at the epicenter of the 6.8 magnitude earthquake (28.50° N, 87.45° E) and the northern foot of the Himalayas in southwestern Tibet.

Spanning a vast expanse, the earthquake-impacted zone exhibits pronounced topographic diversity. In the southern reaches of the county, high-altitude mountainous terrain prevails, dominated by the Himalayan range. This area is characterized by steep gradients and incised valleys. Conversely, the northern sector features gentler plateaus and sedimentary basins, offering a stark contrast in relief.

In the study area, the built-up area is mainly composed of scattered rural settlements, and the building density changes due to the influence of topography and socio-economic conditions. Many buildings are built of stone, wood, and adobe with limited seismic performance and are vulnerable to damage. The complex topography and structural diversity of the region pose challenges to the extraction of damaged buildings based on remote sensing.

### 2.2. Damaged Building Samples

For this research, multi-source data in Table 1 were collected to comprehensively capture the pre- and post-earthquake conditions of the Shigatse earthquake area. High-resolution optical satellite images are crucial for visual inspection of building conditions. The GF-7 and Beijing-3 satellites provided post-earthquake optical images taken on 11 January 2025, shortly after the earthquake. The pre-earthquake satellite images, captured on 16 December 2024, were provided by Google satellite imagery. These images were pre-processed prior to use, including geometric, radiometric, and atmospheric corrections, noise removal, and georeferencing.

The integrity of Open Street Map (OSM) data in the study area was checked based on pre-disaster images. Then, through visual comparison of OSM data and post-earthquake images, damaged buildings shapefiles were delineated. After obtaining the shapefile of damaged buildings, the post-earthquake remote sensing images were segmented into fixed-size patches of 1024 × 1024 pixels. To enhance the model’s generalizability, random data augmentation techniques such as rotation, horizontal flipping, and vertical flipping were applied to the image patches. This process yielded 2353 post-earthquake image samples, forming a robust dataset for model training and evaluation.

### 2.3. Methodologies

#### 2.3.1. Building the Hybrid Model

**Figure 2:**
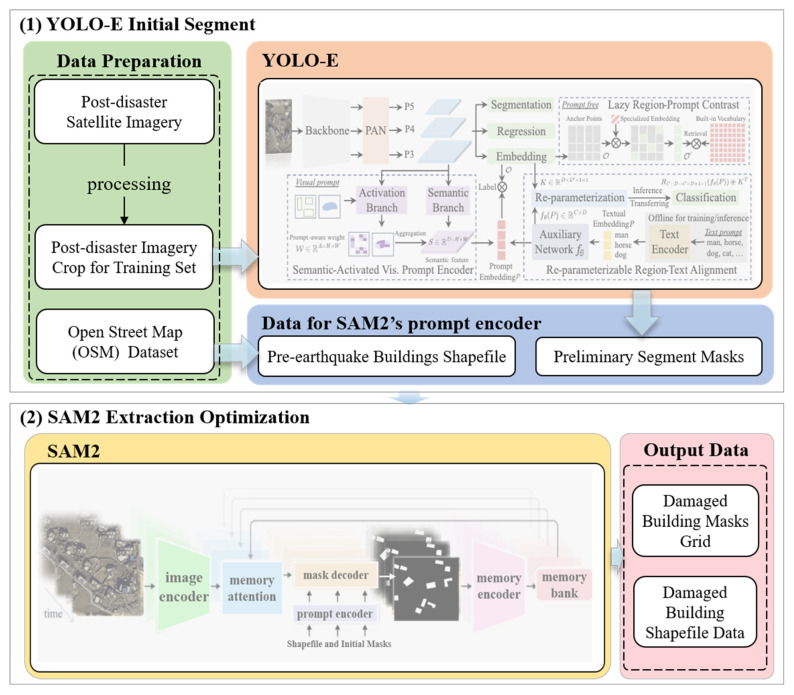
The framework flow chart of the proposed hybrid YOLO-E [26] and SAM2 [27] approach.

As shown in Figure 2, this hybrid model integrates the strengths of YOLO-E and SAM2, aiming to achieve efficient and accurate extraction of earthquake-damaged buildings from remote sensing imagery. In the model architecture, as the preliminary segmentation module, YOLO-E is tasked with swiftly locating and coarsely segmenting potential earthquake-damaged structures in large-scale satellite imagery. With minimal computational demand and reliable feature representation, the model achieves fast inference while maintaining robust performance. Tuning core architectural parameters—such as attention head count and network depth—allows adaptation to the distinct characteristics of post-earthquake imagery. After the initial segmentation, the extracted candidate regions are further refined to enhance segmentation precision by using the SAM2 model. To make the model more suitable for earthquake aftermath scenarios, we fine-tuned the pre-trained SAM2 using remote sensing images from earthquake-affected areas. This helped the model better recognize changes in building textures and shapes. During the model inference stage, we introduced the validated OSM building data as prior knowledge to assist in the identification and extraction of damaged boundaries, thereby ensuring the completeness of the extracted damaged building contours. These files are especially useful when the buildings are badly destroyed or their outlines are difficult to see. Thanks to its boundary-aware design, SAM2 can produce clearer shapes and more reliable segmentations. As a result, the final damage masks show better precision and clearer differences between categories.

#### 2.3.2. Model Training and Optimization

To train the hybrid model, a post-earthquake dataset annotated with precise labels of earthquake-damaged buildings was constructed. The dataset was split into training, validation, and test sets in a 7:2:1 ratio. During training, we used the stochastic gradient descent (SGD) algorithm with an initial learning rate of 0.001 and a momentum of 0.9. To help the model generalize and avoid overfitting, the learning rate was decayed by a factor of 10 every 10 epochs. For the loss function, we combined cross-entropy and mean squared error (MSE): cross-entropy was used for the classification task in YOLO-E to distinguish building presence and damage state, while MSE helped SAM2 align predicted segmentation masks with the ground truth. Validation data were used to track training progress, and we adjusted hyperparameters like batch size and number of epochs based on these results. All experiments were conducted using PyTorch(2.6.0+cu124) on a system with an NVIDIA RTX 4090 GPU (24 GB) and an Intel Xeon Gold 6226 R CPU. The training was repeated iteratively until validation scores—such as precision, recall, F1-score, and IoU—stabilized and the loss function converged to a satisfactory level.

(1) $$Precision=\frac{TP}{TP+FP}$$

(2) $$Recall=\frac{TP}{TP+FN}$$

(3) $$F1-score=\frac{2\times Precision\times Recall}{Precision+Recall}$$

(4) $$IoU=\frac{TP}{TP+FP+FN}$$

where *TP*, *FP*, and *FN* denote true positive, false positive, and false negative, respectively.

## 3. Results and Analysis

### 3.1. Accuracy Evaluation

**Figure 3:**
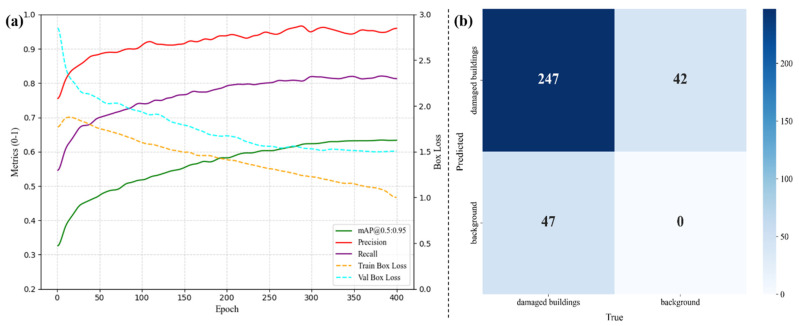
(**a**) Training metrics and box loss for 400 epochs; (**b**) confusion matrix on the test set.

To assess the model’s performance more thoroughly, we first visualized the key precision-related metrics during training, as shown in Figure 3a. Then, we tested the model on 235 images, which included 336 manually labeled damaged buildings. We compared the model’s predictions with the ground-truth labels and used a confusion matrix to evaluate its classification results as shown in Figure 3b. The model achieved a precision of 84.0%, meaning most of the predicted damaged buildings were indeed damaged. The recall was 85.5%, indicating that it correctly detected a large portion of the actual damaged buildings. With an F1-score of 0.847, the model shows a good balance between precision and recall. The IoU score was 0.735, reflecting a fairly strong overlap between the predicted masks and the ground truth.

However, the model was not flawless. Some buildings were misclassified—especially older ones or those under renovation—which had textures similar to damaged structures. Also, some damaged buildings were missed entirely, mostly in areas where trees or debris blocked the view, or where the image quality was poor. Improving detection under these challenging conditions will be an important direction for future work.

### 3.2. Overall Extraction Results

**Figure 4:**
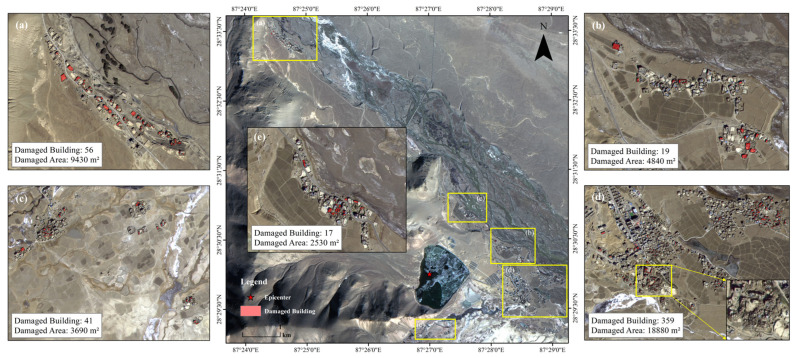
Detected damaged buildings within 20 km of the Shigatse earthquake epicenter. Red polygons mark damage footprints in five typical areas (**a**–**e**), with corresponding statistics shown in each subfigure.

The hybrid model yielded strong performance in extracting damaged buildings in the Shigatse earthquake region. Upon processing post-earthquake satellite imagery, it identified 492 suspected damaged building patches within a 20-km radius from the epicenter in Figure 4. Within a 10-km range, damaged buildings were detected in all surveyed villages. Areas such as Gare Guoji, Xuezhu, Tangren, Chaji, Kangqiong, Jiding, and Jiweng exhibited notable structural damage. The model effectively distinguished damaged from undamaged structures, maintaining clear segmentation boundaries. For instance, in Gare Guoji, collapsed-roof buildings were accurately labeled as damaged, while adjacent intact structures were classified correctly.

Spatial distribution analysis revealed damage clustering along the earthquake fault zone, in agreement with tectonic stress theory. Regions adjacent to the fault typically experience more severe ground motion. Furthermore, damage concentration was higher in low-lying terrain. This may be attributed to seismic wave amplification, which is known to intensify shaking in valleys or depressions. The model successfully captured these trends, demonstrating its capability to generalize across geospatial variations.

### 3.3. Representative Case Analysis

This section conducts a detailed analysis of two representative cases to further evaluate the performance of the hybrid model. These cases correspond to regions D and F, respectively, as defined in Figure 1.

**Figure 5:**
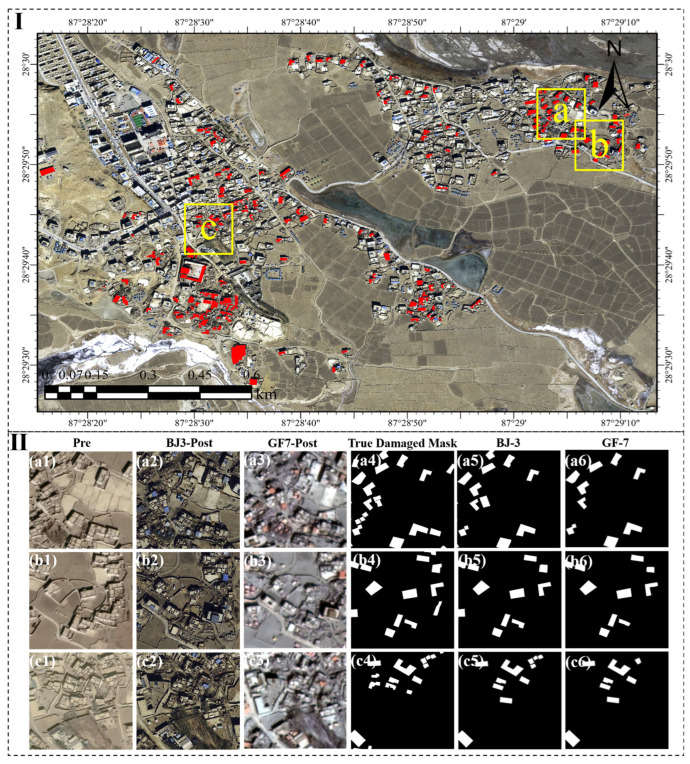
Part I: overview map showing the location of Region D (yellow box) within the study area, with red markers indicating detected damaged buildings. Part II: (**a1**–**c1**) pre-earthquake imagery, (**a2**–**c2**) post-earthquake imagery from Beijing-3 (BJ-3), (**a3**–**c3**) post-earthquake imagery from Gaofen-7 (GF-7), (**a4**–**c4**) ground-truth data of damaged buildings, (**a5**–**c5**) damage extraction results from BJ-3 imagery, and (**a6**–**c6**) damage extraction results from GF-7 imagery.

As shown in Figure 5, Region D, selected for testing, comprised Cuoguo Township (labeled as C) and the Kangqiong area (labeled as A and B), which were further split into subregions a, b, and c. Manually delineated (a4–c4), 46 ground-truth damaged building masks in all were distributed as 19, 13, and 14 masks over the corresponding subregions. With 12, 11, and 11 detections in subregions a, b, and c, respectively, the proposed model on post-earthquake Beijing-3 (BJ-3) imagery extracted 34 damaged buildings overall (a5–c5). With per-subregion accuracies of 63.16%, 84.61%, and 78.57%, this produced an average accuracy of 73.91%. Applying Gaofen-7 (GF-7) post-disaster imagery, the model extracted 28 damaged buildings (a6–c6), with 10, 10, and 8 detections, producing a reduced overall accuracy of 60.87% and subregion accuracies of 52.63%, 76.92%, and 57.14%.

Performance on GF-7 images was limited for continuously aligned damaged buildings (e.g., bottom-left of a4, top-right of c4), according to the results, since the model routinely misclassified adjacent damaged buildings as a single unit. By contrast, BJ-3 images provided better boundary resolution, enabling more exact delineation of individual structures. The model found difficulty, though, with structures displaying irregular or complicated geometric forms across both imagery kinds.

**Figure 6:**
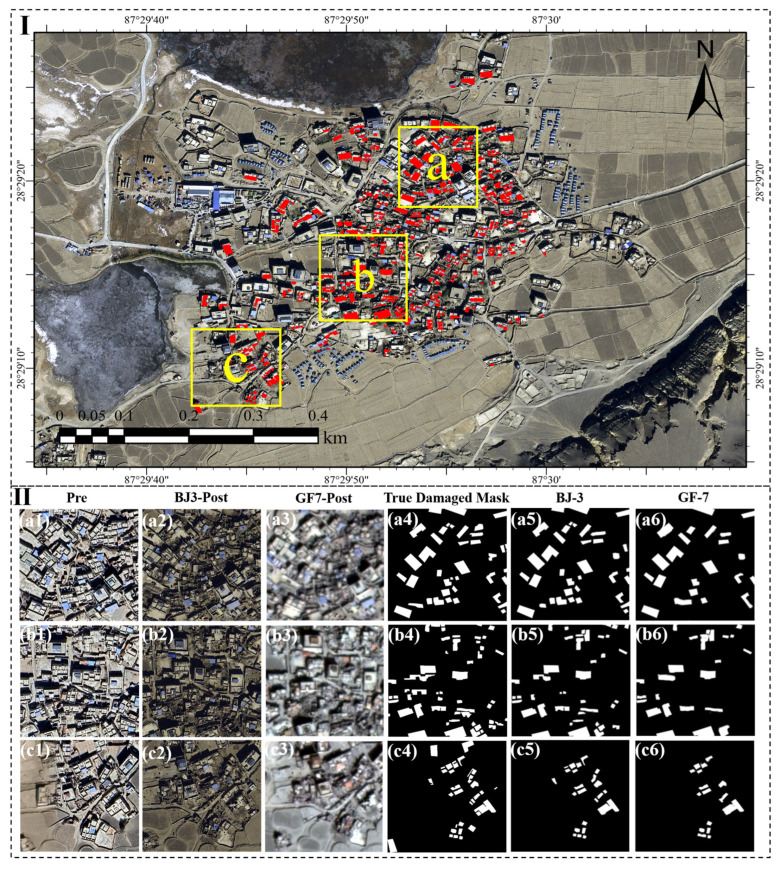
Part I: overview map showing the location of Region F (yellow box) within the study area, with red markers indicating detected damaged buildings. Part II: (**a1**–**c1**) pre-earthquake imagery, (**a2**–**c2**) post-earthquake imagery from Beijing-3 (BJ-3), (**a3**–**c3**) post-earthquake imagery from Gaofen-7 (GF-7), (**a4**–**c4**) ground-truth data of damaged buildings, (**a5**–**c5**) damage extraction results from BJ-3 imagery, and (**a6**–**c6**) damage extraction results from GF-7 imagery.

As shown in Figure 6, Region F, comprising Jiding Village and Jiweng Village, was also divided into subregions a, b, and c, containing a total of 116 ground-truth damaged building masks (a4–c4), specifically 36, 49, and 31 masks, respectively. The application of the model to BJ-3 post-disaster imagery resulted in the detection of 99 buildings, of which 96 were accurately extracted (a5–c5), including 35, 38, and 23 detections in subregions a, b, and c. This leads to an overall accuracy of 82.76%, with subregion accuracies of 97.22%, 77.55%, and 74.19%. In contrast, the analysis of GF-7 imagery detected only 62 buildings (a6–c6), with 22, 24, and 16 detections in each subregion, which resulted in a lower overall accuracy of 53.45%, along with subregion accuracies of 61.11%, 48.98%, and 69.57%. Further analysis shows that in subregions b and c, where damaged buildings are small in scale and densely distributed, the model exhibited moderate performance for both image sources, indicating a need for refinement in detecting compact and clustered damage patterns. Notably, the issue of adjacent building merging observed in Region D using GF-7 imagery was also present in Region F, emphasizing the limitations of GF-7 in high-density damage scenarios.

## 4. Discussion

### 4.1. Comparison with Existing Models

The hybrid model demonstrated high efficacy in extracting earthquake-induced building damage in the Shigatse area. Compared to manual surveys, which are time- and labor-intensive and carry safety risks, the automated approach processes satellite imagery rapidly and safely, offering immediate insights into affected regions.

We benchmarked against U-Net [30], ResU-Net [31], DeepLabV3+ [32], and YOLOv8-Seg [33]—each representing distinct architectures for segmentation. While single-task models often suffer from limited localization accuracy or insufficient segmentation detail, the hybrid model mitigates these shortcomings through a complementary design.

As shown in Table 2, single-task models either lack the ability to accurately localize damaged buildings or struggle with detailed segmentation, while the hybrid model overcomes these limitations by leveraging the complementary strengths of both components. In the table, error count refers to the number of intact buildings that were incorrectly identified as damaged within the six sub-regions described in Section 3.3. With high precision, recall, F1-score, and IoU, and a lower error count, the model effectively handles complex post-earthquake conditions. It can accurately extract damaged buildings, providing critical information for emergency teams to prioritize rescue efforts and providing valuable data for urban planners and policymakers during reconstruction. By quickly extracting the areas most affected by disasters, resources can be allocated more effectively, potentially saving lives and reducing long-term economic losses.

### 4.2. Transferability Test

To evaluate the transferability and robustness of the proposed building damage extraction method, we selected post-earthquake remote sensing datasets from the 2023 earthquakes in Kahramanmaraş and Gaziantep, two of the most severely affected regions in Turkey [34]. These datasets were used for both model training and testing. The post-earthquake imagery, acquired by the Jilin-1 satellite at a high spatial resolution of 0.5 m, predominantly features masonry structures with more uniform and regular geometrical shapes. This structural typology is distinct from the adobe-style, irregularly shaped buildings commonly found in the Shigatse region of Tibet, which were used in the prior experiment area. In addition to the architectural differences, the regional geomorphological conditions in Turkey, characterized by relatively flatter urban terrains and different vegetation coverage, diverge significantly from the complex mountainous landscapes of Dingri County.

**Figure 7:**
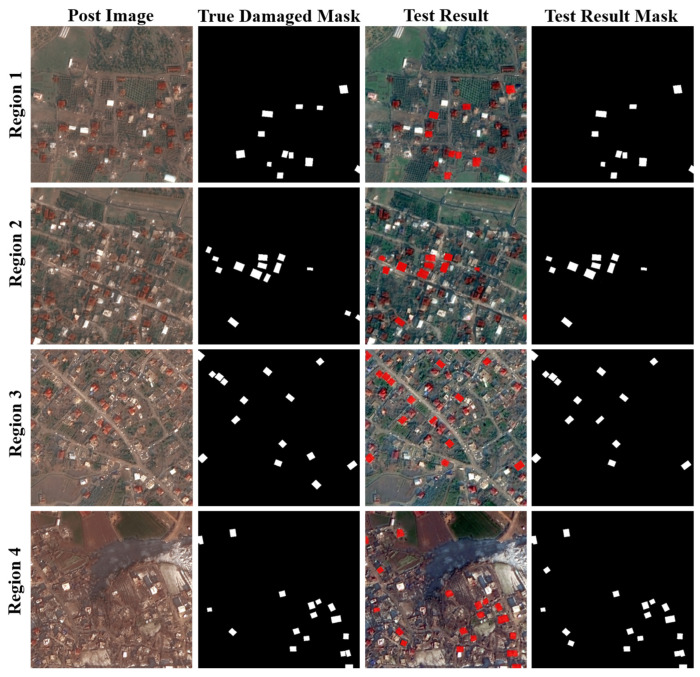
Test results comparison chart.

The testing results across the four selected regions in Figure 7 indicate that, although some cases of missed detections, false positives, and merged adjacent damaged buildings were observed, the overall extraction performance remains satisfactory. These results confirm that the proposed method possesses a certain degree of transferability and generalization capability across different regions.

### 4.3. Limitations and Outlook

Although this study has achieved encouraging results, there are still some limitations. The performance of the proposed hybrid model is highly dependent on the quality of the input data. In areas with low satellite image quality, the accuracy of damaged building extraction often decreases significantly, particularly when the spatial resolution falls below the minimum required threshold of 0.80 m.

In addition, the model tends to misclassify buildings with irregular structures that existed before the earthquake, which indicates that further improvement is needed to distinguish the intrinsic variability of the building from the actual seismic damage. There are also obvious challenges in extracting small and densely distributed damaged buildings. If the post-earthquake image resolution is insufficient, the boundary of the damaged building becomes blurred, which often leads to the wrong merging of adjacent buildings. Such misclassification and merging not only reduce the spatial accuracy of building extraction but also introduce deviations in damage area statistics, thereby compromising the reliability of disaster assessments.

Future research should focus on improving data preprocessing strategies, such as incorporating super-resolution reconstruction models to enhance the spatial resolution of post-disaster remote sensing imagery, thereby improving the accuracy of damaged building extraction. In addition, incorporating earthquake scene datasets from regions with diverse geographical characteristics into the training set can enhance the model’s generalization ability and cross-regional adaptability. Furthermore, integrating advanced network architectures—such as enhanced attention mechanisms or GAN-based image enhancement techniques—may further improve overall model performance.

## 5. Conclusions

This study successfully developed a hybrid model for building damage extraction in the Shigatse earthquake-stricken area. The model combines the real-time detection and segmentation capabilities of YOLO-E and the pixel-level accuracy of SAM2 to achieve high-performance damage building extraction. Through the comprehensive collection and detailed data preprocessing of multi-source data, such as pre-earthquake and post-earthquake satellite images, the model uses labeled post-earthquake image datasets for training. In the training process, the stochastic gradient descent optimization algorithm is used, combined with cross-entropy and mean squared error loss functions. The accuracy of the model is 0.840, the recall rate is 0.855, the F1-score is 0.847, and the IoU is 0.735. The model extracted 492 suspected damaged building patches within a radius of 20-km from the epicenter. The results show that the disasters are mainly concentrated in fault zones and low-lying areas. The model provides a fast and reliable tool for rescue teams to find severely damaged areas and improve rescue effectiveness. Moreover, the proposed method was evaluated on the post-earthquake remote sensing datasets from the 2023 Turkey earthquake to assess its transferability. The experimental results demonstrate that the method achieves satisfactory performance under varying regional conditions, indicating its potential for transferability and generalization.

Future research may focus on the following directions: incorporating super-resolution reconstruction models to enhance image quality, expanding training datasets with greater geographical diversity to improve model generalization, exploring more advanced deep learning architectures to boost performance, and developing real-time disaster monitoring systems for practical applications.

## Figures and Tables

**Table 1:** Details of the remote sensing imagery.

Satellite/Sensor	Acquisition Date	Spatial Resolution	Data Type
GF-7/DLC	11 January 2025	0.80 m(PAN)	Post-earthquake
		3.20 m(MUX)	
Beijing-3/N	11 January 2025	0.30 m	Post-earthquake
Google Satellite	16 December 2024	0.15 m	Pre-earthquake

**Table 2:** Comparison of the different models.

Models	Precision (%)	Recall (%)	F1_Score (%)	IoU (%)	Error Counts
U-Net	83.03	82.11	82.59	70.54	8
ResU-Net	84.06	81.79	82.94	71.13	5
DeepLabV3	83.92	82.76	83.34	71.43	6
YOLOv8-Seg	84.25	84.83	84.54	73.21	4
Proposed model	84.01	85.47	84.73	73.51	4

## Data Availability

The post-earthquake damaged building dataset for Dingri County, Shigatse, Tibet, constructed in this study, has been publicly released and is accessible at https://doi.org/10.57760/sciencedb.26871.
